# Effectiveness of ertugliflozin during Ramadan fasting in patients with type 2 diabetes mellitus: a real-world study (ErtuRamadan study)

**DOI:** 10.3389/fendo.2025.1542946

**Published:** 2025-02-11

**Authors:** Md Faruque Pathan, Nazma Akter, Marufa Mustari, M. Saifuddin, Mirza Sharifuzzaman, Mohammad Motiur Rahman, Mohammed Ripon, S. M. Mohiuddin, A. B. M. Kamrul-Hasan, Mohammad Abdul Hannan, Muhammad Shah Alam, Samira Mahjabeen, Faria Afsana, Muhammed Abu Bakar, Tahniyah Haq, Afsar Ahammed, Samir Kumar Talukder, Sourav Sarkar, Shahjada Selim

**Affiliations:** ^1^ Department of Endocrinology, BIRDEM General Hospital, Dhaka, Bangladesh; ^2^ Department of Endocrinology, MARKS Medical College & Hospital, Dhaka, Bangladesh; ^3^ Department of Endocrinology, Bangabandhu Sheikh Mujib Medical University (BSMMU), Dhaka, Bangladesh; ^4^ Department of Endocrinology, Dhaka Medical College & Hospital, Dhaka, Bangladesh; ^5^ Department of Medicine, Rajshahi Medical College & Hospital, Rajshahi, Bangladesh; ^6^ Department of Endocrinology, Rangamati Medical College & Hospital, Rangamati, Bangladesh; ^7^ Department of Endocrinology, Sir Salimullah Medical College, Dhaka, Bangladesh; ^8^ Department of Endocrinology, Mymensingh Medical College, Mymensingh, Bangladesh; ^9^ Department of Endocrinology, North East Medical College & Hospital, Sylhet, Bangladesh; ^10^ Department of Endocrinology, Army Medical College, Cumilla, Bangladesh; ^11^ Department of Endocrinology, Chattogram Maa O Shishu Hospital Medical College, Chattogram, Bangladesh; ^12^ Department of Endocrinology, National Institute of Traumatology and Orthopaedic Rehabilitation (NITOR), Dhaka, Bangladesh; ^13^ Department of Endocrinology, Rangpur Medical College Hospital, Rangpur, Bangladesh; ^14^ Medicine & Endocrinology, Boalkhali Upazila Health Complex, Chattogram, Bangladesh

**Keywords:** diabetes mellitus, Ramadan, fasting, ertugliflozin, SGLT2 inhibitors, safety, efficacy

## Abstract

**Background:**

Management of type 2 diabetes mellitus (T2DM) during Ramadan fasting presents unique challenges due to prolonged fasting periods, irregular meal schedules, and altered medication timing, potentially impacting glycemic control. Ertugliflozin, a sodium-glucose co-transporter 2 (SGLT2) inhibitor, has been shown to improve glycemic control in T2DM effectively. However, the effectiveness of ertugliflozin during Ramadan fasting, a period with unique glycemic challenges, has not been studied extensively.

**Methods:**

This study was a multicenter, real-life experience study involving 1373 adult patients with known T2DM for at least one year, an HbA1c level of less than 10%, and who intended to fast during Ramadan. Participants were divided into two groups: the Ertu group (n=703), consisting of patients who had been on a stable dose of ertugliflozin for at least three months before Ramadan, and the non-Ertu group (n=670), which included patients receiving other oral antihyperglycemic drugs (OADs) except ertugliflozin. Patients attended a baseline visit one month before the first day of Ramadan and a follow-up visit within one month after the last day of Ramadan. Both visits included history taking, physical examinations, and laboratory tests. The primary endpoints were changes in HbA1c levels, body weight, body mass index (BMI), and the incidence of hypoglycemia during Ramadan fasting.

**Results:**

The mean age of the study participants was 50.37 ± 11.14 (SD) years, with 40.6% male and 58.7% female. Patients receiving ertugliflozin showed significant reduction in HbA1C (-0.65 ± 0.67% vs. -0.22 ± 0.64%, p<0.001), body weight (-1.24 ± 2.58 kg vs. -0.36 ± 3.41 kg, p<0.001), and BMI (-0.48 ± 1.03 kg/m² vs. -0.11 ± 1.33 kg/m², p<0.001) compared to the non-Ertu group. Hypoglycemia was reported in 0.3% of the ertugliflozin group and 0.7% of the other group, with comparable adverse events (p=.23; ≥0.05), indicating a favorable safety profile for ertugliflozin during fasting.

**Conclusion:**

This study demonstrates that ertugliflozin is effective and safe for patients with T2DM during Ramadan fasting.

## Introduction

Fasting during the holy month of Ramadan is an essential component of regular spiritual practice for adult Muslims, requiring abstinence from food and drink for 12 to 20 hours a day. For individuals with diabetes mellitus (DM), this significant alteration of meal times may lead to adverse health consequences including hypoglycemia, hyperglycemia, dehydration, etc. (1–5). Many Muslim patients with type 2 diabetes mellitus (T2DM) choose to fast each year, despite medical advice cautioning against fasting (4–6). Ongoing anti-diabetic medication may also increase potential risks for adverse health conditions if careful attention to choose anti-diabetic agents followed by adjustments in dose and timing is not implemented (7).

A number of studies have been conducted focusing on the efficacy and safety of antidiabetic agents (such as metformin, sulphonyleureas, DPP-4 inhibitors, GLP-1 agonists, and SGLT2 inhibitors) (8–11). DPP-4 inhibitors, metformin, and GLP-1 agonists are generally safer than sulphonylureas and insulin during fasting, with a lower risk of hypoglycemia (12–19). However, recent guidelines have prioritized the use of sodium-glucose co-transporter 2 inhibitors (SGLT2i) for managing diabetes during Ramadan fasting considering their benefits including weight management, cardiovascular benefits, renal protection, and flexibility in dosing, though caution is advised regarding potential hypoglycemia when combined with insulin or sulphonylureas (20, 21). SGLT2 inhibitors, including canagliflozin, dapagliflozin, empagliflozin, and ertugliflozin, have demonstrated effective glycemic control in patients with type 2 diabetes mellitus (T2DM), both as monotherapy and as add-on therapy, along with additional benefits such as weight loss and blood pressure reduction (22, 23). Ertugliflozin, the newest member of this class, received global approval in 2017 as part of a comprehensive treatment plan alongside standard diet and exercise (24). With a daily single-dose oral administration schedule, ertugliflozin alone or in combination has shown benefits in reducing cardiorenal risk, including a 10% reduction in hospitalization for heart failure and approximately a 30% reduction in the progression of kidney disease (25–30). Consequently, ertugliflozin provides a convenient dosing option during Ramadan fasting, offering effective glycemic control and additional health benefits, including weight loss, blood pressure reduction, cardiovascular protection, and preservation of renal function (31). However, due to the risk of dehydration, several guidelines recommend caution in the use of SGLT2 inhibitors for individuals at risk of volume depletion or those taking angiotensin-converting enzyme inhibitors (ACE-I) and diuretics (32).

Several clinical trials and studies have assessed the safety and efficacy of SGLT2 inhibitors during the holy month of Ramadan, reporting low risks for hypoglycemia, dehydration, postural hypotension, diabetic ketoacidosis, or genitourinary infections with these drugs (8, 11, 33–38). Trial on empagliflozin found showed a significantly low frequency of hypoglycemia (16%) compared to the standard treatment group (32%) concluding stating this drug with a lower risk of hypoglycemia symptoms and higher tolerability (36). A trial on canagliflozin showed 92% of patients experienced no hypoglycemic events during Ramadan, maintaining a consistent safety profile (38). A study on dapagliflozin found that 3.4% of patients reported symptomatic hypoglycemia compared to 19.2% in the sulphonyleurea group, resulting in a 76% relative risk reduction for hypoglycemia in patients taking dapagliflozin during the 4th week of Ramadan (35).

The effectiveness of ertugliflozin during Ramadan fasting remains underexplored, particularly in real-world settings where patient characteristics, adherence to fasting practices, and dietary habits can vary widely. Current studies have primarily focused on other SGLT2 inhibitors, leaving a gap in understanding how ertugliflozin specifically affects glycemic control and safety outcomes for Muslim patients with T2DM during fasting. With the rising incidence of diabetes, especially in Bangladesh where Muslims are predominant, research on diabetes management during Ramadan is crucial. A similar study conducted in Bangladesh evaluated the efficacy and safety of empagliflozin in patients with T2DM during Ramadan and observed significant improvements in HbA1c and weight reduction, with no serious adverse events reported (34). This study, conducted in a population with similar ethnic and cultural lifestyles, demonstrated that empagliflozin was both effective and safe for use during fasting periods. These findings provide a useful reference point for further studies on ertugliflozin in similar contexts. Thus, the ErtuRamadan study assessed the effectiveness of ertugliflozin among T2DM patients who fast during Ramadan. The results will help healthcare providers create targeted, evidence-based treatment plans and enhance decision-making for Muslim T2DM patients observing fast.

## Methods

### Subjects and study design

ErtuRamadan study was a real-life experience study conducted over a period of five months (January 2024 to May 2024). Fifteen specialized centers of different divisions of Bangladesh dedicated to the treatment of diabetes mellitus were considered study sites.

Muslim patients aged ≥ 18 years with T2DM for at least 1 year, an HbA1c <10%, who intended to observe Ramadan fasting, and who had been on stable doses of ertugliflozin for at least 3 months before Ramadan (either as monotherapy or in combination with other anti-hyperglycemic agents) were approached for inclusion in this study. Patients with any acute condition, and taking insulin or any other SGLT2 inhibitors within 3 months prior to include in the study, having contraindication for ertugliflozin were excluded from this study.

Participants were enrolled in two groups based on whether they were receiving ertugliflozin as OAD or not. The patients receiving ertugliflozin along with or without other OADs were categorized as the Ertu group. The patients receiving standard care with any other OADs were categorized as non-Ertu group. Patients receiving insulin were excluded from this study.

**Figure 1:**
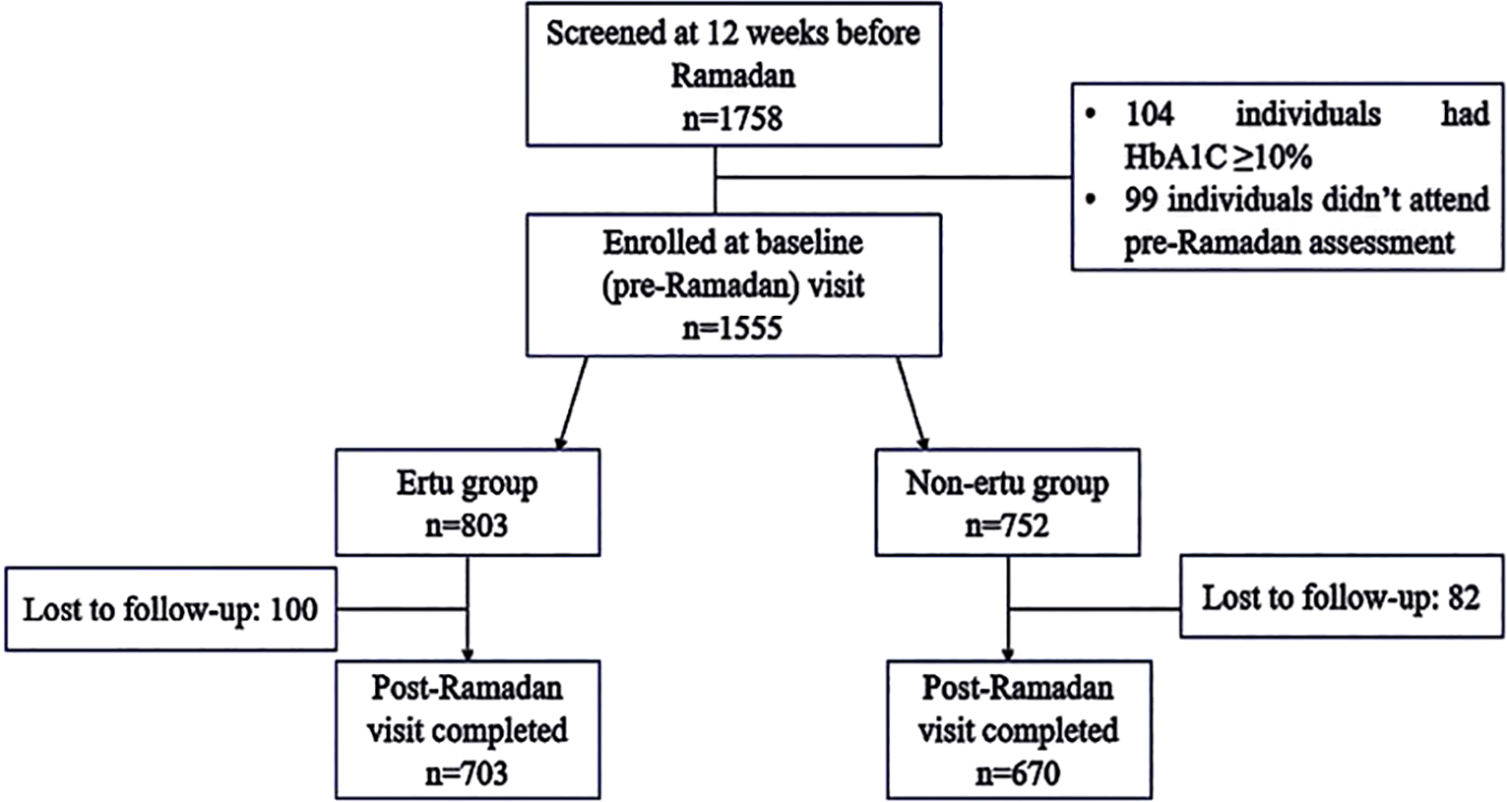
Patient disposition in ErtuRamadan study.

A total of 1373 patients were included in final analysis where the Ertu group included 703 patients and non-Ertu group included 670 patients (**Figure 1**). As this study was designed as a real-world observational study, the treatment allocation (Ertugliflozin vs. other OHA) was conducted based on the physician’s clinical judgment. No matching for baseline glycemic status was performed, as the aim was to evaluate the effectiveness of Ertugliflozin in routine clinical practice.

Throughout the month of Ramadan, all patients in the Ertu group continued their ertugliflozin treatment, while those in the non-Ertu group were not initiated ertugliflozin or any other SGLT2 inhibitors. All participants were required to attend a screening and enrollment visit three months prior to Ramadan, as well as baseline and follow-up visits before within 1 month, during, and after 1 month of Ramadan for clinical assessment and data collection. Informed written consent was obtained from each patient before enrollment.

### Study end-points and assessment

The primary end point of the study was the change from baseline (pre-Ramadan assessment) in HbA1c level. Secondary end points included the proportion of patients achieving the desired glycemic control (HbA1c<7.0%), changes from baseline in fasting plasma glucose, post-prandial plasma glucose, systolic and diastolic blood pressure, eGFR, serum creatinine, serum sodium (Na) and potassium (K) and incidence of other adverse events (AEs) related to ertugliflozin (urinary tract infections, genital mycotic infection, and osmotic diuresis–related AEs) or incidents of other illness not related to ertugliflozin during Ramadan.

### Study procedure

Data were collected using a semi-structured case record form, which included relevant information regarding sociodemographic profiles, anthropometric measurements, and clinical information from the enrolled patients. Each participant underwent a comprehensive history taking, physical examination, and necessary laboratory investigations.

During the study, patients maintained a logbook for 30 days to record their blood glucose levels, instances of hypoglycemia (symptomatic or reported), adverse events (symptomatic or reported), number of fasting days, and any changes in medication. They were instructed to utilize their logbooks to report any hypoglycemic events immediately. In cases of reported hypoglycemia, adjustments to medications were made as necessary based on regular assessments conducted from the logbook entries.

After Ramadan, patients were evaluated for changes in body weight and relevant clinical investigations. The logbooks and adverse event forms were collected during this final post-Ramadan visit. Ongoing communication via telephone was also established to monitor patients’ conditions effectively.

Measurements taken included body weight, height, blood pressure, HbA1c, fasting and post-prandial glucose levels, creatinine, sodium (Na), potassium (K), alanine aminotransferase (ALT), and estimated glomerular filtration rate (eGFR), both before and after Ramadan. All data were documented in separate case record forms (CRF).

### Operational definition

Body mass index (BMI): BMI was calculated by dividing the participant’s weight in kilograms by the square of their height in meters, applying the formula kg/m². According to the Asia-Pacific classification defined by the Western Pacific Regional Office of the WHO, BMI categories were as follows: underweight (<18.50 kg/m²), normal (18.50–22.99 kg/m²), overweight (23.00–24.99 kg/m²), and obese (≥25 kg/m²) (39).

Hypoglycemia: Hypoglycemia was defined as plasma glucose levels below 70 mg/dL (<3.9 mmol/L). Severe hypoglycemia was described as episodes involving altered mental or physical functioning necessitating external assistance for recovery. Hypoglycemic episodes were categorized into: symptomatic (with signs such as dizziness, blurred vision, palpitations, nausea, sweating, confusion, tremors, or intense hunger, regardless of biochemical confirmation), biochemically confirmed (self-monitored blood glucose <3.9 mmol/L, with or without symptoms), and severe episodes (requiring external help, or associated with seizures or loss of consciousness) (40).

Volume depletion: This condition was defined as a significant reduction in extracellular fluid volume due to sustained salt and fluid loss exceeding intake. Clinical manifestations included symptoms such as hypotension, orthostatic hypotension, postural dizziness, dehydration, syncope, or presyncope (41).

Urinary tract infection (UTI): A UTI was identified as an infection affecting any component of the urinary system, including the kidneys, ureters, bladder, or urethra, with most cases involving the lower urinary tract (42).

Hypertension: Hypertension is defined as a systolic blood pressure (SBP) of ≥140 mmHg and/or a diastolic blood pressure (DBP) of ≥90 mmHg, measured on at least two separate occasions, or current use of antihypertensive medication by registered physician (43, 44).

Dyslipidemia: Dyslipidemia is defined as abnormal levels of lipids in the blood, typically characterized by elevated total cholesterol (≥200 mg/dL), LDL cholesterol (≥140 mg/dL), triglycerides (≥150 mg/dL), or low HDL cholesterol (<40 mg/dL) (45).

Chronic kidney disease (CKD): CKD is defined as a gradual loss of kidney function over time. It is diagnosed when there is evidence of kidney damage or an estimated glomerular filtration rate (eGFR) below 60 mL/min/1.73 m² for at least three months (46).

Hyponatremia: A serum sodium level of less than 135 milliequivalents per liter (mEq/L) was defined as hyponatremia (47).

Hypokalemia: A serum potassium level of less than 3.5 milliequivalents per liter (mEq/L) was defined as hypokalemia (48).

estimated Glomerular Filtration Rate (eGFR): eGFR is determined using the CKD-EPI (Chronic Kidney Disease Epidemiology Collaboration) equation, which incorporates serum creatinine, age and gender (49).

The formula used is:


$$eGFR=141\times min{\left(Scr/\kappa ,1\right)}^{\alpha }\times max{\left(Scr/\kappa ,1\right)}^{-1.209}\times 0.993^{age}\times \;\left(1.018\;if\;female,\;else\;1\right)$$

- Scr: Serum creatinine (measured in mg/dL).
- κ: 0.7 for females and 0.9 for males.
- α: -0.329 for females and -0.411 for males.
- Age: The patient’s age in years.

### Statistical analysis

Data analysis was conducted with the statistical software SPSS version 25.0. Descriptive statistics was used to describe the baseline characteristics of the study patients. Continuous variables were presented by mean and standard deviation and the categorical variables were presented by frequency and percentages. Association between categorical variables were determined using Chi-square test and Fishers’ exact test according to the applicability. The difference between continuous variables of two groups was determined by an independent student t-test. The difference of continuous variables between pre-Ramadan and post-Ramadan assessment were determined using paired t-test. Statistical significance was considered with a p value of less than 05.

### Ethical consideration

The study protocol was reviewed and approved by the institutional review board (IRB) of Bangabandhu Sheikh Mujib Medical University (BSMMU/2023/4977).

## Result

A total of 1373 patients were included in ErtuRamadan study, 703 patients were receiving ertugliflozin (Ertu group) and 670 were receiving any other anti-hyperglycemic agents than ertugliflozin (non-Ertu group). Demographic and biochemical characteristics of patients at baseline were mostly similar between the two groups except for duration of DM and body weight.

The average age of the participants was 50 years, with 40.6% male and 58.7% female. Notably, the duration of diabetes was significantly longer in the ertugliflozin group compared to the non-Ertu (p <0.05). In terms of body weight, patients in the ertugliflozin group had a higher average compared to those in the non-Ertu group (p <0.05). Regarding comorbidities, both groups exhibited high rates of hypertension and dyslipidemia, but the ertugliflozin group had a significantly higher prevalence of fatty liver disease and coronary artery disease (p <0.05) (**Table 1A**).

**Table 1A:** Baseline demographic characteristics of study participants (n= 1373).

	Total n=1373 n (%)	Ertu group n=703 n (%)	Non-Ertu group n=670 n (%)	*p* value
**Age (years))[mean ± SD]**	50.37 ± 11.14	50.21 ± 11.11	50.53 ± 11.17	0.59*
Gender				
Male	558 (40.6)	276 (39.7)	281 (42.1)	0.379*
Female	806 (58.7)	419 (60.3)	387 (57.9)	
**Duration of diagnosis of DM (years)^⸙^**	6.26 ± 4.98	6.74 ± 5.39	5.75 ± 4.46	<0.001**
Anthropometric measurements^⸙^				
Body weight (kg)	69.34 ± 11.07	69.93 ± 11.54	68.74 ± 10.53	0.04*
BMI (kg/m^2^)	26.78 ± 6.21	26.85 ± 6.24	26.74 ± 6.11	0.73**
Blood pressure (mmHg)^⸙^				
Systolic blood pressure	128.11 ± 23.01	127.90 ± 29.14	128.33 ± 13.95	0.74**
Diastolic blood pressure	80.06 ± 18.76	80.03 ± 24.93	80.08 ± 8.42	0.96**
Comorbidities and complications				
Hypertension	958 (69.8)	479 (68.2)	479 (71.5)	0.18*
Dyslipidemia	954 (69.5)	481 (68.5)	472 (70.4)	0.43*
Fatty liver disease	496 (36.1)	223 (31.8)	273 (40.7)	0.001*
Neuropathy	355 (25.9)	182 (25.9)	173 (25.8)	0.96*
Bronchial asthma	232 (16.9)	106 (15.1)	126 (18.8)	0.06*
CKD	85 (6.2)	41 (5.8)	43 (6.4)	0.66*
Coronary artery disease	73 (5.3)	29 (4.1)	44 (6.6)	0.04*
COPD	52 (3.8)	28 (4)	24 (3.6)	0.69*
Stroke	43 (3.1)	18 (2.6)	25 (3.7)	0.21*
Peripheral artery disease	29 (1.4)	11 (1.6)	8 (1.2)	0.55*
Heart failure	12 (.9)	5 (.7)	7 (1)	0.50*

Data presented as mean ± standard deviation^⸙^ and frequency (percentage) as appropriate.

DM, diabetes mellitus; BMI, body mass index; CKD, Chronic Kidney Disease; COPD, Chronic Obstructive Pulmonary Disease.

*p* value was determined by chi-square test* and independent student t test.**.

Missing data excluded.

Glycemic control was poorer in the Ertu group, with an average HbA1c of 8.16% compared to 7.79% in the non-Ertu group (p <0.05). The estimated glomerular filtration rate (eGFR) and serum creatinine level was comparable across both groups. Serum ALT was also similar in both groups. Regarding serum electrolytes, serum Na was similar in both groups but serum K was lower in patients of non-Ertu group (p<.05) (**Table 1B**).

**Table 1B:** Baseline biochemical characteristics of study participants (n= 1373).

	Total n=1373 n (%)	Ertu group n=703 n (%)	Non-Ertu group n=670 n (%)	*p* value
Glycemic status^⸙^				
HbA1C (%)	7.98 ± .91	8.16 ± .91	7.79 ± .87	<0.001**
FPG (mmol/l)	8.71 ± 1.89	8.85 ± 1.82	8.58 ± 1.94	0.010**
Post prandial PG (mmol/l)	12.10 ± 2.84	12.22 ± 2.77	11.97 ± 2.90	0.114**
Renal function related parameter^⸙^				
eGFR (mL/min/1.73 m²)	83.53 ± 21.64	84.11 ± 21.96	82.95 ± 21.25	0.43**
Serum creatinine (mg/dl)	.88 ± .39	.87 ± .46	.89 ± .30	0.30**
**Serum ALT (U/L)**	38.39 ± 12.06	38.31 ± 11.62	38.48 ± 12.52	0.79**
Serum electrolytes^⸙^				
Serum Na (mEq/L)	138.52 ± 3.54	138.56 ± 3.44	138.47 ± 3.67	0.67**
Serum K (mEq/L)	3.90 ± .46	3.93 ± .48	3.86 ± .43	0.01**

Data presented as mean ± standard deviation^⸙^ and frequency (percentage) as appropriate.

FPG, Fasting Plasma Glucose; Post prandial PG, Postprandial Plasma Glucose (mg/dl); eGFR, Estimated Glomerular Filtration Rate; Serum ALT, Serum Alanine Aminotransferase; Serum Na, Serum Sodium; Serum K, Serum Potassium; Serum creatinine.

*p* value was determined by chi-square test* and independent student t test**.

Missing data excluded.

**Figure 2:**
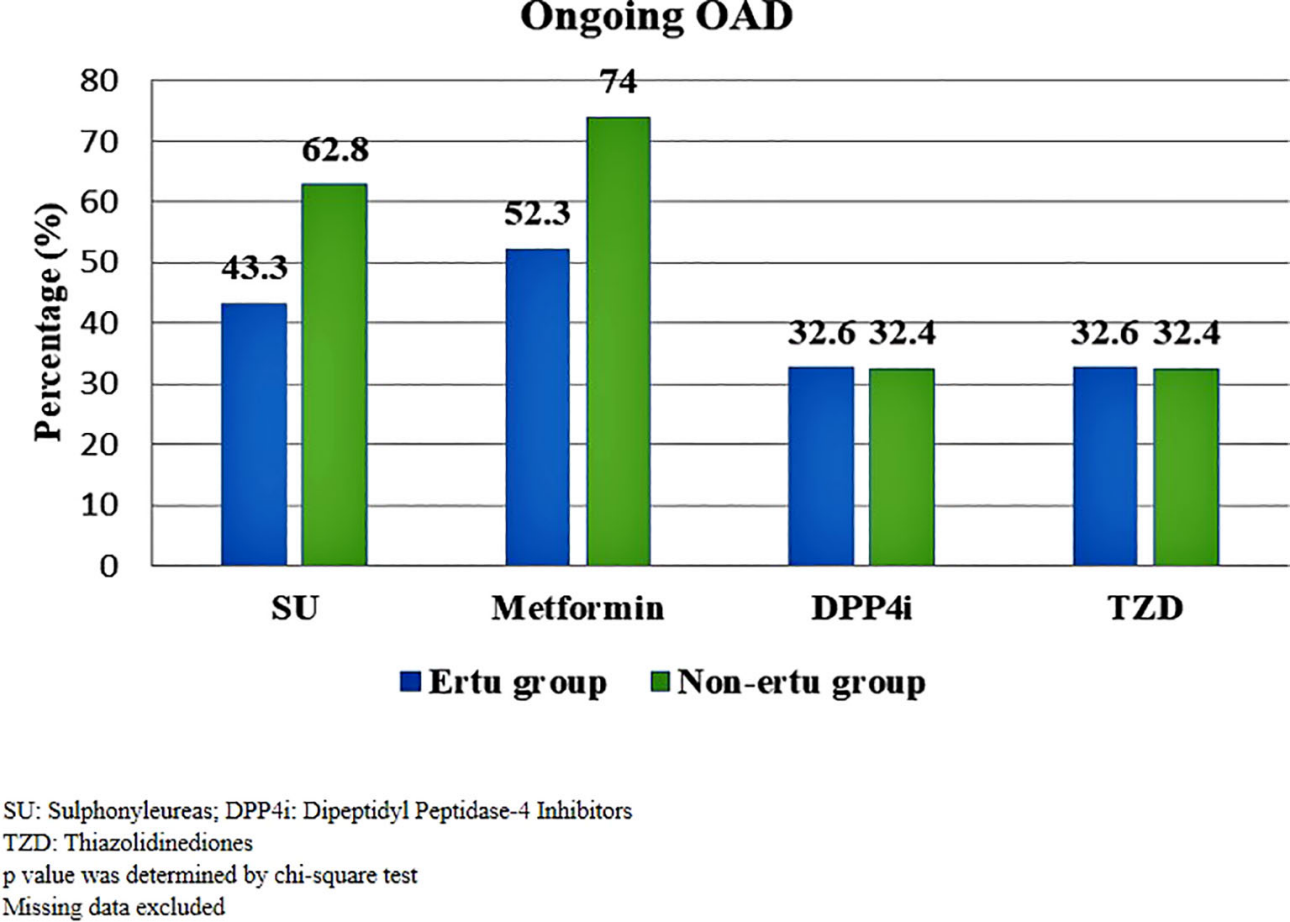
Ongoing oral anti-diabetic medications among study participants (n=1373).

A significantly greater proportion of patients in the non-Ertu group were on sulphonyleureas (62.8% vs. 43.3%) and metformin (74% vs. 52.3%) compared to the Ertu group (p < 0.001). However, the use of DPP4 inhibitors (32.6% vs. 32.4%) and thiazolidinediones (32.6% vs. 32.4%) was similar across both groups (**Figure 2**).

**Figure 3:**
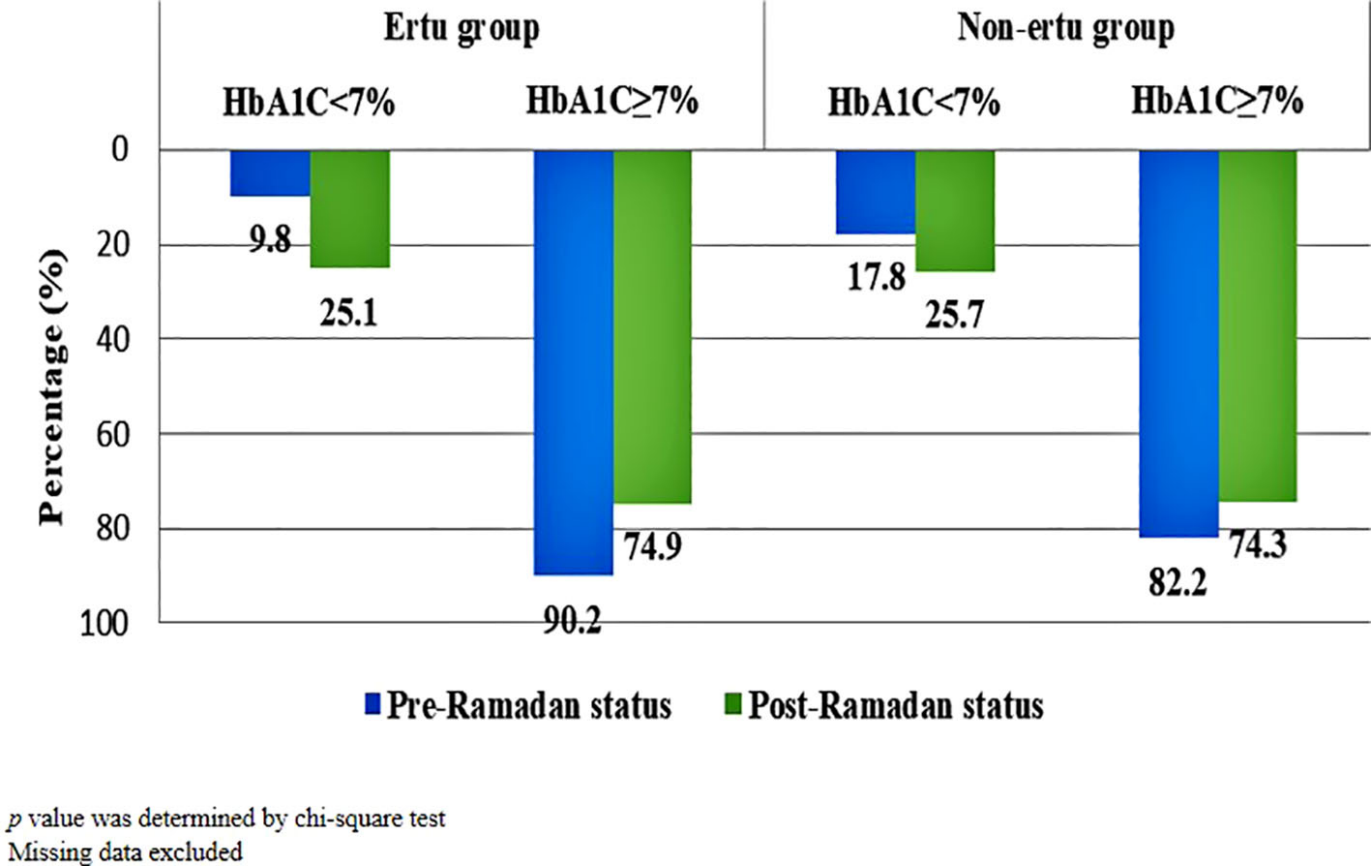
Pre-Ramadan and post-Ramadan glycemic control (HbA1C) status of study participants (n=1373).

Both the ertugliflozin and non-ertugliflozin groups showed significant improvements in glycemic control after Ramadan (p<0.001). In the ertugliflozin group, the proportion of patients achieving good glycemic control (HbA1c<7%) increased from 9.8% to 25.1%, while in the non-ertugliflozin group, it rose from 17.8% to 25.7% (**Figure 3**).

**Figure 4:**
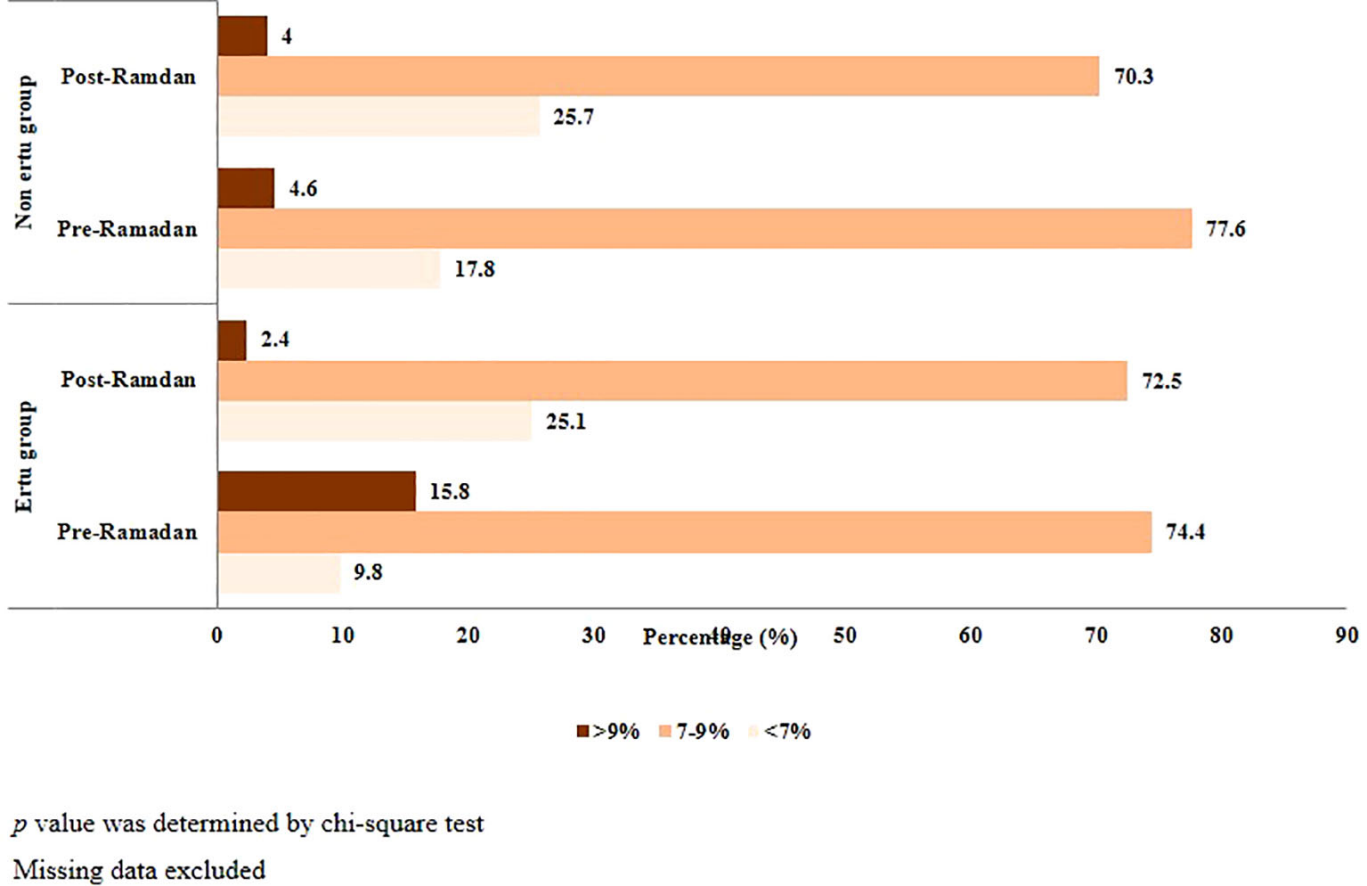
Pre-Ramadan and post-Ramadan HbA1C levels of study participants (n=1373).

The ertugliflozin group demonstrated a more pronounced improvement in glycemic control compared to the non-ertugliflozin group (**Figure 4**).

HbA1c was significantly reduced from pre-Ramadan to post-Ramadan in both groups. Similarly, fasting plasma glucose (FPG) levels and postprandial plasma glucose (PPG) were significantly reduced in both groups. In terms of serum electrolytes, serum Na levels significantly decreased in both groups irrespective of diuretics or ACE inhibitors medication (p < 0.001). SerumK levels also showed slight significant reduction in the Ertu group, while the non-Ertu group saw an increase with statistical significance (**Table 2**).

**Table 2:** Pre- and post-Ramadan glycemic parameters and serum electrolytes in study participants (n=1373).

	Ertu group n=703			Non-Ertu group n=670		
	Pre-Ramadan	Post-Ramadan	p* value	Pre-Ramadan	Post-Ramadan	p* value
MeanHbA1C	8.16 ± .91	7.50 ± .81	<.001	7.79 ± .87	7.57 ± .99	<0.001
FPG	8.85 ± 1.83	7.19 ± 1.03	<.001	8.6 ± 1.95	7.91 ± 1.97	<0.001
PPG	12.20 ± 2.76	9.11 ± 1.45	<.001	12.01 ± 2.89	10.52 ± 2.92	<0.001
Serum Na level	138.56 ± 3.44	138.08 ± 3.08	<.001	139.47 ± 3.67	138.92 ± 3.26	<0.001
Serum K level	3.93 ± .43	3.88 ± .37	<.001	3.86 ± .43	3.91 ± .28	<0.001

Data presented as mean ± standard deviation.

*p* value was determined by paired t test*.

In the ertugliflozin group, patients experienced significant reductions in both body weight and BMI compared to the non-Ertu group (p < 0.05). Glycemic control was also notably better in the ertugliflozin group, with significant improvements observed in HbA1c, fasting plasma glucose, and post-prandial plasma glucose levels (p < 0.05 for all comparisons). Both systolic and diastolic blood pressure reductions were more pronounced in the ertugliflozin group than in the non-Ertu group (p < 0.05). Additionally, serum sodium and potassium levels showed significant decreases in the ertugliflozin group, while these levels increased in the non-Ertu group (p < 0.05). However, no significant differences were observed in the changes in estimated glomerular filtration rate (eGFR) between the two groups (**Table 3**).

**Table 3:** Change in blood glucose parameter, body weight, BMI, blood pressure, eGFR, and other biochemical values from baseline at post-Ramadan visit among study participants (n=1373).

	Ertu group n=703 Mean ± SD	Non-Ertu group n=670 Mean ± SD	p value
Change in body weight (kg)	-1.24 ± 2.58	-.36 ± 3.41	<0.001
Change in BMI (kg/m^2^)	-.48 ± 1.03	-.11 ± 1.33	<0.001
Change in systolic blood pressure (mmHg)	-5.76 ± 11.35	-4.33 ± 11.49	0.030
Change in diastolic blood pressure (mmHg)	-2.76 ± 8.18	-1.64 ± 8.83	0.030
Change in HbA1C (%)	-.65 ± .67	-.22 ± .64	<0.001
Change in FPG (mmol/l)	-1.66 ± 1.43	-.68 ± 1.57	<0.001
Change in Post prandial PG (mmol/l)	-3.09 ± 2.23	-1.48 ± 2.47	<0.001
Change in eGFR (mL/min/1.73 m²)	7.30 ± 9.03	7.97 ± 11.91	0.380
Change in ALT (U/L)	-2.73 ± 6.79	-1.61 ± 7.95	0.008
Change in Na (mEq/L)	-.52 ± 2.97	.47 ± 3.55	<0.001
Change in K (mEq/L)	-.04 ± .32	.04 ± .39	<0.001

Change in parameter = (Post-Ramadan assessment value) ─ (Pre-Ramadan assessment value).

p value was determined by independent student t test.

In the ertugliflozin group, significant reductions in BMI were observed in the overweight and obese categories, with a notable decrease post-Ramadan. The normal-weight and underweight categories did not show significant changes. In contrast, the non-ertugliflozin group showed minimal changes in BMI across all categories, except for obese category (**Table 4**).

**Table 4:** Pre- and post-Ramadan BMI of the study participants across different BMI categories (n=1373).

BMI category	BMI					
	Ertu group n=703			Non-Ertu group n=670		
	Pre-Ramadan	Post-Ramadan	p*value	Pre-Ramadan	Post-Ramadan	p*value
	mean ± SD	mean ± SD		mean ± SD	mean ± SD	
Underweight	17.41 ± .50	17.16 ± .83	0.156	17.41 ± 1.45	17.82 ± 2.20	0.58
Normal	21.57 ± 1.10	21.54 ± 1.47	0.734	21.55 ± 1.45	21.84 ± 1.91	0.09
Overweight	23.92 ± .62	23.56 ± 1.14	0.003	24.08 ± .59	24.18 ± 1.22	0.291
Obese	29.35 ± 4.20	28.73 ± 4.10	<0.001	29.60 ± 3.83	29.33 ± 3.91	<0.001

p value was determined by paired t test.

**Figure 5:**
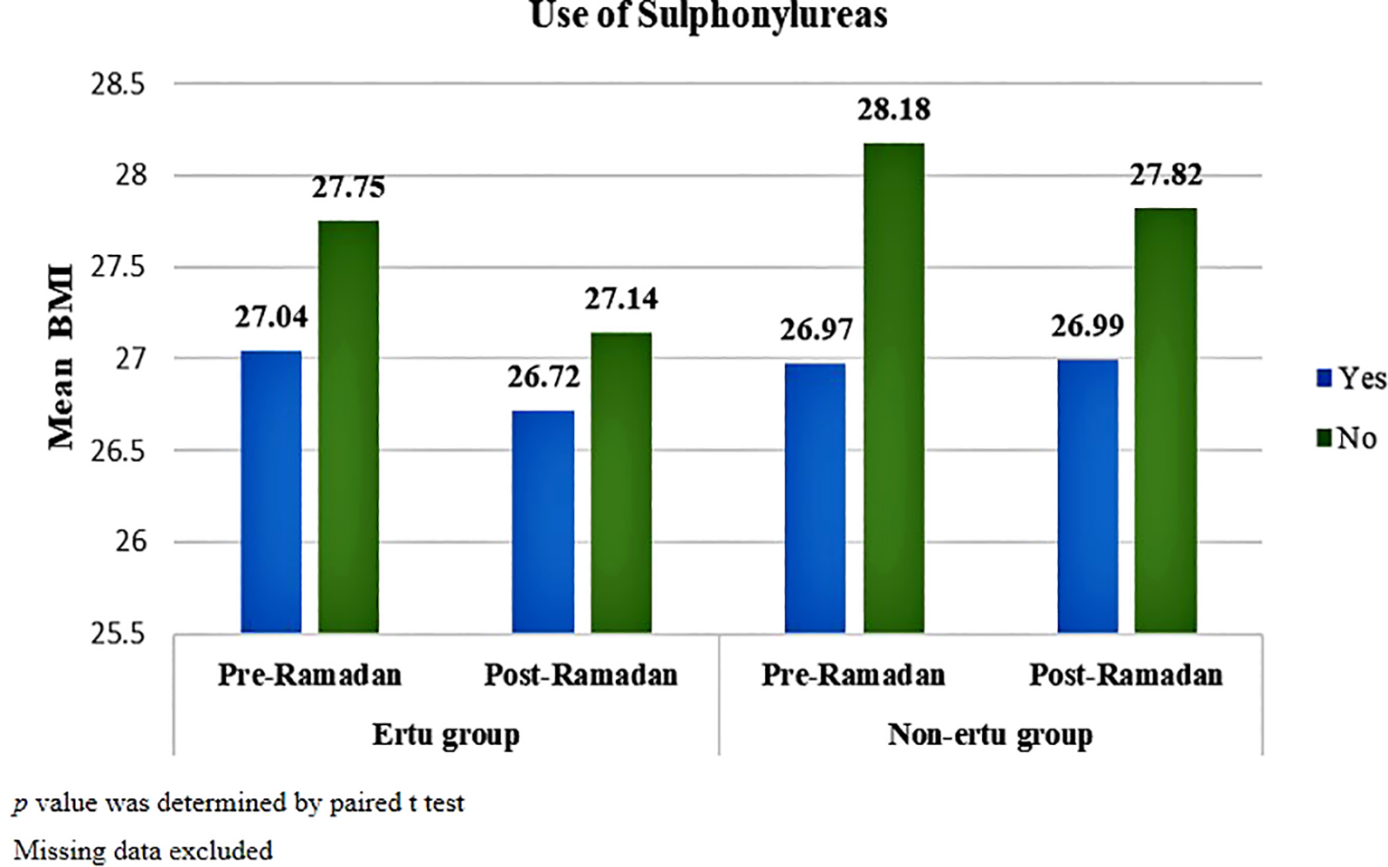
Pre-and post-Ramadan BMI in study participants stratified by use of sulphonylureas (n=1373).

In both groups, both patients who received and didn’t receive sulphonylureas showed statistically significant changes in BMI after Ramadan (p<001). But patients not using sulphonylureas had a more pronounced decrease in BMI post-Ramadan compared to those using sulphonylureas, with the Ertu group showing more consistent reductions than the non-Ertu group. p value was determined by paired t test (**Figure 5**).

Significant reductions in HbA1c were observed in most BMI categories for both groups. In the Ertu group, normal-weight, overweight, and obese patients had significant decrease of BMI while underweight patients showed a non-significant reduction. In the non-Ertu group, all BMI categories, including underweight patients, experienced significant HbA1c reductions post-Ramadan. Overall, the Ertu group showed more pronounced decreases in HbA1c across most categories (**Table 5**).

**Table 5:** Pre- and post-Ramadan HbA1Camong the study participants across different BMI categories (n=1373).

BMI category	HbA1C					
	Ertu group n=703			Non-Ertu group n=670		
	Pre-Ramadan	Post-Ramadan	p* value	Pre-Ramadan	Post-Ramadan	p*value
	mean ± SD	mean ± SD		mean ± SD	mean ± SD	
Underweight	7.95 ± 1.89	7.45 ± 1.48	0.08	8.05 ± 1.20	7.75 ± 1.06	0.004
Normal	8.24 ± .81	7.52 ± .72	<0.001	7.71 ± .91	7.50 ± .93	<0.001
Overweight	8.15 ± .81	7.49 ± .72	<0.001	7.76 ± .95	7.68 ± 1.15	<0.001
Obese	8.16 ± .94	7.51 ± .84	<0.001	7.82 ± .84	7.56 ± .94	<0.001

p value was determined by paired t test.

Hypoglycemia was reported in 0.3% patients of Ertu group and 0.7% patients of non-Ertu group. Incidents of urinary tract infections, abdominal pain, and dehydration were low and not statistically different between groups. Overall, adverse events were comparable (p≥0.05) (**Table 6**).

**Table 6:** Adverse events during Ramadan (n=1373).

Adverse events	Ertu group n (%)	Non-Ertu group n (%)	p value
UTI	2 (.3)	3 (.4)	0.61**
Abdominal pain	4 (.6)	2 (.3)	0.45**
Dehydration	4 (.6)	6 (.9)	0.47**
GI symptoms	10 (1.4)	16 (2.4)	0.18*
Heartburn	11 (1.6)	16 (2.4)	0.27*
Hypoglycemia	2 (.3)	5 (.7)	0.23**
Hypotension	1 (.1)	0	0.51**
Syncope/dizziness/vertigo	4 (.6)	0	0.06**

p value was determined by chi-square test* and Fisher’s exact** test as appropriate.

## Discussion

Ertugliflozin, an SGLT2 inhibitor, has demonstrated considerable efficacy in the management of type 2 diabetes mellitus (T2DM) in previous trials (25–30). Its’ insulin-independent mechanism leads to significant glycosuria and osmotic diuresis, resulting in improved glycemic control (50). Additionally, ertugliflozin contributes to the reduction of cardio-renal risks in patients with T2DM (51). However, concerns about the safety and efficacy of SGLT2 inhibitors during prolonged Ramadan fasting prompted this clinical trial to assess the effectiveness of ertugliflozin in T2DM patients observing Ramadan in Bangladesh (32). This study observed effective glycemic control and comparable safety in patients receiving ertugliflozin (Ertu group) as monotherapy or add-on with other OAD.

At baseline, patients of both arms exhibited similar demographic characteristics. The mean age of participants was 50 years which aligns with previous national studies (52–54). A higher proportion of female participants were observed with a male: female ratio of 1:1.44 which aligns with previous research on T2DM patients in Bangladesh (34, 52). However, it contrasts with other studies in the country where male participants generally outnumber females (55, 56), potentially due to cultural and social factors. Most participants were urban residents. The average BMI was 26 which lies upper than normal value. Moreover, the majority of the patients were presented with a considerable burden of comorbidities while hypertension and dyslipidemia were the most common. These findings are consistent with existing literature indicating that individuals with T2DM often present with multiple health challenges that complicate their management during periods of fasting (57–60).

In baseline or pre-Ramadan assessment, only 9.8% of patients in the Ertu group and 17.9% patients in non-Ertu group were presented with a controlled glycemic status (<7%) with an overall mean of 7.98 ± .91 (SD)%. Previous studies conducted in Bangladesh reported nearly similar mean HbA1C (34, 61). The mean HbA1C was 8.16 ± .91 (SD)% and 7.79 ± .87 (SD)% in Ertu group and non-Ertu group accordingly where HbA1C was statistically higher in Ertu group. The higher baseline HbA1c and lower number of patients with glycemic control observed in the Ertu group reflects a poorer initial glycemic control, likely due to longer diabetes duration and associated comorbidities. The post-Ramadan assessment demonstrated that patients of both arms experienced a significant reduction in HbA1C levels. Patients with good glycemic control were statistically increased in post-Ramadan assessment to 25.1% in the Ertu group and 25.7% in non-Ertu group. These findings coordinate with previous studies where Ramadan fasting and ongoing management leads to a statistically significant reduction in blood glucose levels of T2DM patients (62–64). The change in HbA1C from pre-Ramadan assessment to post-Ramadan assessment is significantly higher in the ertugliflozin arm compared to control arm (-.65 ± .67% Vs -.22 ± .64%). This finding underscores ertugliflozin’s effectiveness in glycemic control during fasting. Previous study conducted in Bangladesh focusing on the role of empagliflozin, another SGLT2 inhibitor in T2DM patients observing Ramadan fasting was reported similar findings (34). Other SGLT2 inhibitors including canagliflozin, dapagliflozin and empagliflozin also showed significant improvement in glycemic status during Ramadan fasting (11, 35, 36, 65). An overall reduction in body weight and BMI was observed which was significantly pronounced in the ertugliflozin group, highlighting its role in weight management for overweight individuals with T2DM. Similar findings were reported by canagliflozin and empagliflozin (34, 65). But another study reported weight and BMI reduction in Ramadan fasting regardless of whether patients were on SGLT2 inhibitors or not (37).

However, electrolyte changes were also observed, particularly in the ertugliflozin group. Serum sodium and potassium levels decreased significantly post-Ramadan in the ertugliflozin group, with sodium reducing from 138.56 to 138.08 mEq/L and potassium from 3.93 to 3.88 mEq/L. In contrast, the non-ertugliflozin group showed a slight increase in potassium levels while sodium levels also decreased but to a lesser extent. These findings highlight the diuretic effect of ertugliflozin, which may contribute to mild electrolyte imbalances, emphasizing the need for careful monitoring of electrolytes during fasting periods to prevent dehydration and related complications.

In the present study, comparable rates of hypoglycemic episodes were observed in patients taking ertugliflozin and those on other anti-hyperglycemic agents (0.3% vs. 0.7%). These findings align with previous research indicating that SGLT2 inhibitors are generally associated with fewer hypoglycemic events compared to traditional therapies (11, 35, 36, 65). The few hypoglycemic events were also mild symptomatic in nature. Patients were instructed to record events immediately, enabling healthcare providers to monitor and respond effectively. No severe hypoglycemic events requiring extensive medical intervention were reported. All cases were successfully managed through standard home-based interventions and no dose or medication adjustment or alteration was required. Furthermore, the study revealed no statistically significant differences in the rates of dehydration between the groups, indicating that ertugliflozin can be safely administered during fasting periods. The risk of volume depletion is particularly pertinent during Ramadan fasting, as prolonged periods without food or water may lead to dehydration, postural hypotension, and dizziness. However, our study did not observe significant adverse effects related to volume depletion in the ertugliflozin group.

The study was not beyond limitations. The first limitation was that this study excluded patients receiving insulin. A large number of patients use insulin along with or without SGLT2 inhibitors during Ramadan fasting, highlighting the need for further study on those receiving insulin. Another limitation was that we could not consider the dose and duration of the other OADs and stratify accordingly. Another limitation was that there was a possibility of recall bias regarding self-reported symptoms.

These findings of this study support the use of ertugliflozin as an effective and safe option for managing T2DM in patients observing Ramadan fasting. By demonstrating significant improvements in glycemic control and a favorable safety profile, ertugliflozin offers a treatment option for Muslim individuals with T2DM during Ramadan. Further research is warranted to confirm these findings and explore the long-term safety and efficacy of SGLT2 inhibitors during prolonged fasting periods.

## Conclusion

In individuals with T2DM fasting during Ramadan, the use of Ertugliflozin is safe, effective, and well-tolerated. Patients experienced significant improvements in glycemic control, with a notable reduction in HbA1c levels. Additionally, reductions in body weight and BMI were also more pronounced in the ertugliflozin group compared to non-ertugliflozin users. However, a thorough pre-Ramadan assessment and education, including hydration advice, are essential followed by careful monitoring is essential. Further structured clinical trials should be conducted to support this finding.

## Data Availability

The raw data supporting the conclusions of this article will be made available by the authors, without undue reservation.
